# Effect of Light Scattering and Higher-order Aberrations on Visual Performance in Eyes with Granular Corneal Dystrophy

**DOI:** 10.1038/srep24677

**Published:** 2016-04-18

**Authors:** Kazutaka Kamiya, Hidenaga Kobashi, Akihito Igarashi, Nobuyuki Shoji, Kimiya Shimizu

**Affiliations:** 1 Department of Ophthalmology, University of Kitasato School of Medicine, Japan; 2 Kitasato University School of Allied Health Sciences, Kanagawa, Japan

## Abstract

This study was aimed to assess the relationship of intraocular forward scattering, corneal backward scattering, and corneal higher-order aberrations (HOAs) with corrected distance visual acuity (CDVA) in eyes with granular corneal dystrophy (GCD). We retrospectively examined forty two eyes of 42 consecutive patients who diagnosed GCD, and age-matched 20 eyes of 20 healthy subjects. We assessed objective scattering index (OSI) using the double-pass instrument (OQAS II, Visiometrics), corneal densitometry (CD) using the Scheimpflug rotating camera (Pentacam HR, Oculus), and corneal HOAs using the Hartmann-Shack aberrometry (KR-9000, Topcon). The OSI, CD, and corneal HOAs were significantly larger in the GCD group than those in the control group (Mann-Whitney U test, p < 0.001). We found significant correlations of logMAR CDVA with the OSI (Spearman correlation coefficient r = 0.577, p < 0.001), and with the CD (r = 0.340, p = 0.028), but no significant association with corneal HOAs (r = 0.061, p = 0.701). Intraocular forward scattering, corneal backward scattering, and corneal HOAs in eyes with GCD were higher than that in normal eyes. The CDVA was significantly correlated with intraocular forward scattering, but not with corneal HOAs in eyes with GCD, suggesting that light scattering, especially forward scattering, plays a more vital role in visual performance than corneal aberrations in eyes with GCD.

Granular corneal dystrophy (GCD) is an autosomal-dominant, bilateral, noninflammatory corneal disorder characterized by the deposition of discrete, irregularly shaped, grayish-white opacities in the anterior to middle central portion of the cornea. The progressive accumulation of the deposits with aging in this corneal disorder may deteriorate corneal transparency in the axial area, and subsequent visual performance for such patients. Nevertheless, the degree and the morphology of the corneal deposits varies among different patients with GCD, especially in homozygotes^1^,^2^,^3^,^4^,^5^. Indeed, we encountered some patients whose corrected distance visual acuity (CDVA) was not good, even when the granular depositions of the cornea was mild by slit-lamp microscopy and the area of corneal stroma between the deposits remained clear in a clinical setting. This deterioration of visual performance may be attributed to the induction of light scattering and/or higher-order aberrations (HOAs) by the cornea. However, there have so far been only a few studies on light scattering or HOAs in eyes with GCD. In addition, all these studies have been focused merely on corneal backward scattering, which does not necessarily reflect visual performance for such patients in daily practice^6^,^7^. The purpose of the present study is twofold: to compare the values of the forward and backward scatterings and HOAs for GCD with those for age-matched healthy subjects, and to assess the relationships of the forward and backward scatterings and corneal HOAs with CDVA in eyes having GCD.

## Results

**Figure 1:**
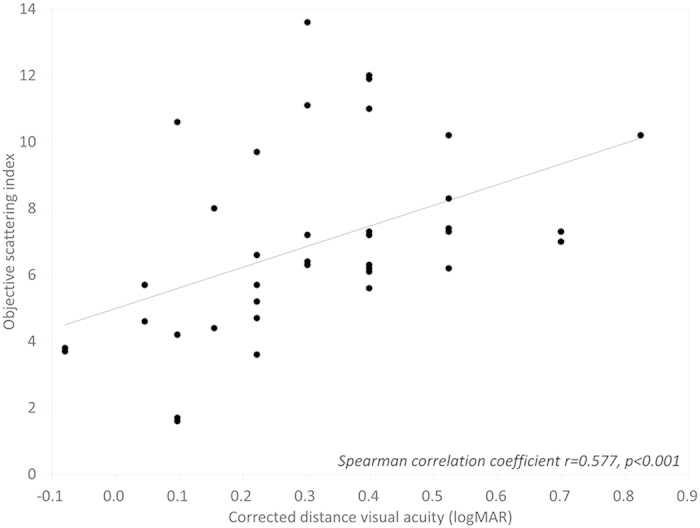
A graph showing a significant correlation between logarithm of the minimal angle of resolution corrected distance visual acuity and objective scattering index (Spearman correlation coefficient r = 0.577, p < 0.001).

**Figure 2:**
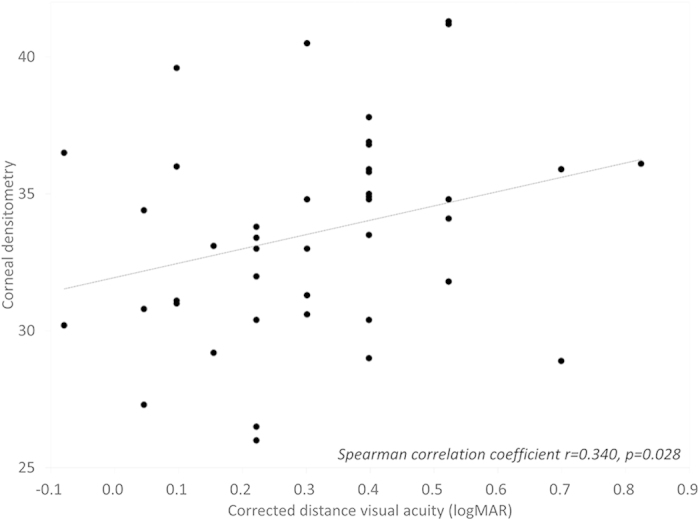
A graph showing a significant correlation between logarithm of the minimal angle of resolution corrected distance visual acuity and corneal densitometry (Spearman correlation coefficient r = 0.340, p = 0.028).

**Figure 3:**
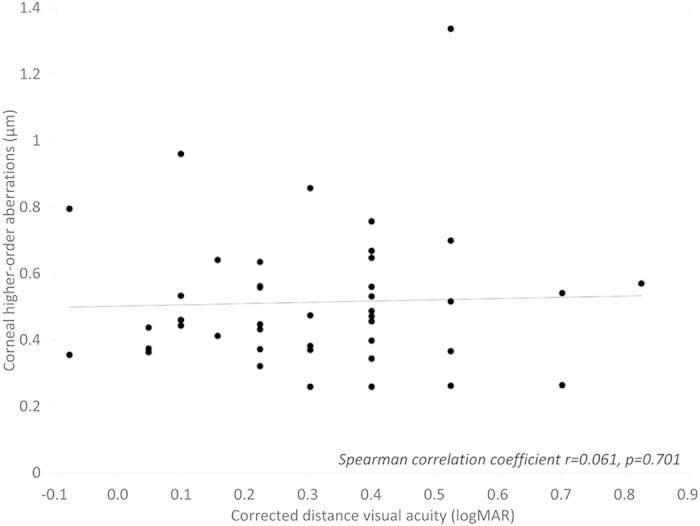
A graph showing no significant correlation between logarithm of the minimal angle of resolution corrected distance visual acuity and corneal higher-order aberrations (Spearman correlation coefficient r = 0.061, p = 0.701).

The patient demographics of the study population are listed in Table 1. The mean objective scattering index (OSI), corneal densitometry (CD), corneal HOAs, and logarithm of the minimal angle of resolution (logMAR) CDVA were 6.91 ± 2.72, 33.56 ± 3.72 GSU, 0.51 ± 0.21 μm, and 0.31 ± 0.20, respectively, in the GCD group. The corresponding figures were 1.53 ± 0.91, 18.70 ± 1.54 GSU, 0.19 ± 0.11 μm, and −0.11 ± 0.06, respectively, in the control group. We found significant differences in OSI, CD, corneal HOAs, or logMAR CDVA between the two groups (Mann-Whitney U test, p < 0.001). We found significant correlations of logMAR CDVA with the OSI (Spearman correlation coefficient r = 0.577, p < 0.001)(Fig. 1), and with the CD (r = 0.340, p = 0.028) in the GCD group (Fig. 2). On the other hand, we found no significant association of logMAR CDVA with corneal HOAs in the GCD group (r = 0.061, p = 0.701) (Fig. 3).

## Discussion

In the current study, our results demonstrated that CDVA was significantly associated with the OSI and the CD, but not with corneal HOAs in the GCD group, suggesting that intraocular forward scattering as well as corneal backward scattering plays a more essential role in visual performance for GCD than corneal HOAs. Until now, there have been only a few studies performing detailed analysis of HOAs or light scattering in eyes with GCD, and all previous studies have focused on the localization of corneal backward scattering^6^,^7^. As far as we can ascertain, this is the first study to o perform the detailed analysis of visual performance such as light scattering and aberrations in eyes with GCD.

With regard to the forward scattering, this is also the first study to objectively assess the forward scattering, which may more precisely reflect visual performance than the backward scattering, in eyes with GCD. Although the exact etiology of this forward scattering remains unclear, we assume that the scattering is largely derived from the deposits of the anterior to middle portion of the cornea, because no eyes had concomitant eye diseases such as clinically significant cataract, posterior capsular opacification, macular atrophy, epiretinal membrane, vitreous opacification, uveitis, severe glaucoma, or ocular trauma, in this series. Hong *et al.* demonstrated using the Fourier domain optical coherence tomography (OCT) that the linear deposits were located most deeply in the cornea, followed by granular deposits and diffuse haze moving anteriorly in eyes with GCD^8^. We previously demonstrated that the OSI was significantly associated with visual performance not only in normal eyes^9^, but also in post-descemet’s stripping automated endothelial keratoplasty eyes^10^. We consider that the objective assessment of forward scattering is of clinical help for predicting visual performance even in eyes with GCD.

With regard to the backward scattering, Kim *et al.* showed using densitometry line profiles that discrete granules were surrounded by relatively clear areas in eyes with GCD type 2^6^. Kocluk *et al.* demonstrated using the Scheimpflug camera that corneal density at the apex in macular corneal dystrophy group was significantly higher than that in granular or lattice corneal dystrophy group^7^. Mori *et al.* showed that three-dimensional OCT could visualize the reduction of the area of the opacity with progressively deeper simulated ablation to determine the appropriate ablation depth in eyes with GCD^11^. Kim *et al.* also described that Fourier domain OCT provided depth of the deposits for GCD quite precisely, allowing the determination of the appropriate depth of phototherapeutic keratectomy and the appropriate selection of lamellar versus penetrating keratoplasty as the procedure of choice^12^. It is indicated that both anterior segment OCTs were useful for objective preoperative and noninvasive assessments of corneal opacities for GCD. However, no quantitative relationship of the corneal opacities with visual performance has been so far elucidated. We believe that the CD, as a measure of the backward scattering of the cornea by the Scheimpflug camera, reflects, to some extent, visual performance for GCD.

Our results also demonstrated that the OSI, CD, and corneal HOAs in the GCD group were significantly larger than those in control group, suggesting that not only light scattering but also aberration in eyes with GCD was significantly higher than that in normal eyes. Although corneal HOAs play more subtle role in visual performance than light scattering in eyes with GCD, it should be noted that corneal HOAs can be another source of deteriorating visual performance especially in GCD eyes requiring phototherapeutic keratectomy for the surgical removal of the diseased tissue.

There are several limitations to this study. Firstly, the sample size in this study was relatively small, and the study design was retrospective. Although the sample size in this study offered >78% statistical power at the 5% level, a prospective study with a large number of patients is necessary to confirm our preliminary findings. Secondly, the possible source of the intraocular forward scattering cannot be identified. However, we assume that the origin of this forward scattering is largely derived from the granular deposits of the cornea, since no concomitant eye diseases exist in the study population. Thirdly, we did not confirm the reproducibility of the corneal HOA measurements in eyes with GCD. We cannot deny the possibility that the accuracy and repeatability of the Hartmann-Shack aberrometry might be decreased by the presence of corneal dense deposits.

In conclusion, our pilot study supports the view that CDVA was significantly correlated with the forward scattering of the eye and the backward scattering of the cornea, but not with corneal HOAs, in eyes having GCD. It suggests that the intraocular forward scattering as well as corneal backward scattering plays a more important role in visual performance than corneal HOAs for GCD in a clinical setting. We believe that this information is clinically helpful for understanding the detailed etiology of deteriorating visual performance for GCD.

## Methods

### Study Population

A total of forty two eyes of 42 consecutive patients (10 men and 32 women), who diagnosed GCD by one experienced examiner (KK), were included in this retrospective observational study, as the study group (GCD group). Any concomitant eye diseases such as clinically significant cataract, posterior capsular opacification, macular atrophy, epiretinal membrane, vitreous opacification, uveitis, severe glaucoma, and trauma, were excluded from the study. Age-matched 20 eyes of 20 ophthalmologically normal population, who had no ophthalmic disease other than refractive errors, were also included in our study as the control group. The sample size in this study offered 99.3% statistical power at the 5% level in order to detect a correlation of 0.58, and offered 78.5% statistical power at the 5% level in order to detect a correlation of 0.35. This retrospective review of the clinical charts was approved by the Institutional Review Board at Kitasato University and was performed in accordance with the Declaration of Helsinki. Our Institutional Review Board waived the requirement for informed consent for this retrospective study. Patient data was anonymized before access and/or analysis.

### Assessment of Visual Acuity, Forward and Backward Scattering and Higher-order Aberrations

We quantitatively assessed logMAR CDVA, OSI, CD, and corneal HOAs, in both groups. Visual acuity measurement was performed using a Snellen chart with Japanese letters at a distance of 5 m with best correction (but not with habitual correction).

The OSI, as a measure of the forward scattering of the eye, was determined with Optical Quality Analysis System^TM^ (Visiometrics, Terrassa, Spain) for a 4.0-mm pupil, as described previously. The OSI is an objective evaluation of intraocular scattered light, and the index is calculated by evaluating the amount of light outside the double-pass retinal intensity PSF image in relation to the amount of light on the center. In the particular case of the instrument OQAS, the central area selected was a circle of a radius of 1 minute of arc, while the peripheral zone was a ring set between 12 and 20 minutes of arc^13^. The OSI for normal eyes would range around 1, while values over 5 would represent highly scattered systems. The manifest refractive error of the subjects was fully corrected during these measurements, because the uncorrected refractive error directly affects the optical outcome of the system.

The CD, as a measure of the backward scattering of the cornea, was determined with the rotating Scheimpflug camera (Pentacam HR, Oculus, Germany), as described previously^14^. The Scheimpflug camera provides an image of the whole cornea as well as an objective measurement of the corneal densitometry. The CD was expressed in grayscale units (GSU), and this scale is calibrated by proprietary software, which defines a minimum light scatter of 0 (maximum transparency) and maximum light scatter of 100 (minimum transparency). We used the scale for the whole layers of the cornea within a 2-mm central zone from the corneal apex.

Corneal HOAs were also measured with the Hartmann-Shack aberrometry (KR-9000, Topcon, Tokyo, Japan) for a 4-mm pupil, as described previously^10^. The root-mean-square of the third-order Zernike coefficients was utilized to represent third-order aberrations, the root-mean-square of the fourth-order coefficient to represent fourth-order aberrations. Total HOAs were calculated as the root-mean-square of the third- and fourth-order coefficients.

We performed at least 3 measurements in each device, and the averaged values were used for statistical analysis. The room illumination was kept low (approximately 25 lux) during testing. All examinations were performed by experienced certified orthoptists.

### Statistical Analysis

All statistical analyses were performed using a commercially available statistical software (Ekuseru-Toukei 2010, Social Survey Research Information Co, Ltd., Tokyo, Japan). The normality of all data samples was first checked by the Kolmogorov-Smirnov test. Since the data did not fulfill the criteria for normal distribution, the Spearman correlation coefficient was calculated to assess the relationships of OSI, CD, and HOAs with logMAR CDVA. The Mann-Whitney U test was used to compare the data between the two groups. Unless otherwise indicated, the results are expressed as mean ± standard deviation (SD), a value of p < 0.05 was considered statistically significant.

## Additional Information

**How to cite this article**: Kamiya, K. *et al.* Effect of Light Scattering and Higher-order Aberrations on Visual Performance in Eyes with Granular Corneal Dystrophy. *Sci. Rep.* **6**, 24677; doi: 10.1038/srep24677 (2016).

## Figures and Tables

**Table 1:** Demographics of the study population in eyes with granular dystrophy and normal eyes.

	GCD group	control group	P value
Age (years)	64.9 ± 11.4 years (range, 38 to 82 years)	64.2 ± 9.9 years (range, 39 to 76 years)	0.673
OSI	6.91 ± 2.72 (range, 1.6 to 13.6)	1.53 ± 0.91 (range, 0.7 to 3.4)	p < 0.001
CD	33.56 ± 3.72 GSU (range, 26.0 to 41.3 GSU)	18.70 ± 1.54 GSU (range, 16.6 to 21.7 GSU)	p < 0.001
Corneal HOAs	0.51 ± 0.21 μm (range, 0.26 to 1.34 μm)	0.19 ± 0.11 μm (range, 0.06 to 0.44 μm)	p < 0.001
LogMAR CDVA	0.31 ± 0.20 (range, −0.08 to 0.82)	−0.11 ± 0.06 (range, −0.30 to −0.08)	p < 0.001

GCD = granular corneal dystrophy, OSI = objective scattering index, CD = corneal densitometry, GSU = grayscale units, HOAs = higher-order aberrations, LogMAR = logarithm of the minimal angle of resolution, CDVA = corrected distance visual acuity.
